# Retraction: Chilinda, Z.B., et al. Optimal Household Water Access Fosters the Attainment of Minimum Dietary Diversity among Children Aged 6–23 Months in Malawi. *Nutrients* 2021, *13*, 178

**DOI:** 10.3390/nu13041078

**Published:** 2021-03-26

**Authors:** Zizwani Brian Chilinda, Mark L. Wahlqvist, Meei-Shyuan Lee, Yi-Chen Huang

**Affiliations:** 1 Graduate Institute of Public Health, China Medical University, 91 Hsueh-Shih Road, North District, Taichung City 40402, Taiwan; bryanchilinda@yahoo.co.uk; 2 Department of Nutrition, China Medical University, 91 Hsueh-Shih Road, North District, Taichung City 40402, Taiwan; mark.wahlqvist@gmail.com; 3 National Defense Medical Center, School of Public Health, No.161, Section 6, Minquan East Road, Neihu District, Taipei City 11490, Taiwan; mmsl@ndmctsgh.edu.tw; 4 Institute of Population Health Sciences, National Health Research Institutes, 35 Keyan Road, Zhunan, Miaoli County 35053, Taiwan; 5 Monash Asia Institute, Monash University, 5th Floor, H Building, 900 Dandenong Road, Caulfield East, Melbourne, VIC 3145, Australia

The article cited above [1] has been retracted due to an overlap with a previously published article. 

Following publication, concerns were brought to the attention of the publisher. The article was reassessed in collaboration with the Editors-in-Chief of the journal. The authors agreed to retract the article and apologize for any inconvenience caused by the removal of this article.
